# AI-associated academic writing anxiety in AI-assisted contexts: evidence from Chinese EFL postgraduate students

**DOI:** 10.3389/fpsyg.2026.1841607

**Published:** 2026-06-17

**Authors:** Yaoqin Zhang

**Affiliations:** Department of Foreign Languages, Anhui University of Science and Technology, Huainan, China

**Keywords:** AI-assisted writing, EFL learners, self-efficacy, technology cognition, writing anxiety

## Abstract

**Introduction:**

AI adoption in academic writing provides support but also introduces uncertainty for L2 learners. Research has focused on tool effectiveness and acceptance, with limited attention to how technology perceptions influence emotional experiences, particularly writing anxiety.

**Methods:**

This study proposes a Technology-Emotion-Performance (TEP) framework, integrating the Technology Acceptance Model, social cognitive theory, and appraisal-based emotion theory. Technology appraisals (perceived usefulness, ease of use, trust), writing self-efficacy (perceived control), and writing anxiety (appraisal-based outcome) were examined. Survey data were collected from Chinese EFL postgraduate students, and structural equation modeling (SEM) analyzed the relationships.

**Results:**

Positive technology perceptions were linked to higher writing self-efficacy, which predicted lower writing anxiety. Trust had both direct and indirect effects on anxiety, whereas integrity-related threats increased anxiety. Writing self-efficacy and anxiety were associated with self-reported writing performance.

**Discussion:**

Findings indicate that writing anxiety in AI-assisted contexts is better understood as a technology-mediated appraisal outcome rather than a direct effect of AI use. Integrating cognitive, affective, and behavioral dimensions offers a clearer account of learner experiences and informs pedagogical strategies for AI-supported academic writing.

## 1 Introduction

### 1.1 Background information

The large-scale deployment of generative artificial intelligence (AI) systems in higher education has fundamentally reshaped academic writing practices (Li and Hafner, 2022; Shi and Aubrey, 2025; Mtotywa et al., 2026). Tools powered by large language models now support idea generation, linguistic refinement, structural organization, and citation management in real time (Kasneci et al., 2023). In second language (L2) academic contexts, such technologies reduce linguistic barriers and accelerate drafting processes, thereby altering both the cognitive load distribution and temporal dynamics of writing (Warschauer and Grimes, 2008; Zhang and Hyland, 2018; Wang and Wang, 2025). Academic writing is increasingly becoming a human–AI co-production activity rather than a purely individual cognitive endeavor (Stevenson and Phakiti, 2019; Ramadhan et al., 2024; Rahim and Gregory, 2025).

Educational technology research has primarily examined AI-assisted writing from perspectives of performance enhancement, feedback efficiency, and learner engagement (Li and Hafner, 2022; Yao and Fan, 2025). Studies report improvements in surface-level accuracy (Shi and Aubrey, 2025; Ding et al., 2025; Barrot, 2023), drafting speed (Wang et al., 2024; Xu and Jumaat, 2024), and revision quality (AlAfnan et al., 2023). However, as AI tools shift from post-editing assistants to real-time co-authors, the affective architecture of writing may also be reconfigured (Sheeba Sisly et al., 2026). The automation of linguistic execution and structural generation introduces new evaluative tensions: concerns over originality, dependency and authorship integrity, and the potential erosion of disciplinary voice (Wang and Wang, 2025). These tensions suggest that AI-supported writing environments may generate distinct affective responses that cannot be fully explained by traditional second language writing anxiety frameworks (Wang and Wang, 2025), and learners are no longer solely negotiating language proficiency, they are simultaneously appraising system reliability, algorithmic opacity, ethical risk, and perceived skill displacement (Cheng, 2004; Nugraheni, 2023). The affective consequences of such technology-mediated appraisal processes remain under-theorized and empirically under-modeled, which suggests a broader limitation in current adoption models. The Technology Acceptance Model (TAM) explains technology use through perceived usefulness and perceived ease of use, but it does not sufficiently account for affective disruptions emerging from human–AI co-creation. Conversely, emotion-focused models explain anxiety formation but rarely integrate technological cognition variables into a unified structural framework. As AI tools become embedded in high-stakes academic writing, understanding how cognitive evaluations of AI systems translate into emotional outcomes—and how these emotions subsequently influence performance—becomes theoretically and practically urgent.

To address this gap, the present study reconceptualizes AI-associated academic writing anxiety as a system-level construct emerging from dynamic human–technology interaction. Rather than treating anxiety as an isolated affective variable, this study situates it within an integrated analytical framework that encompasses technology perceptions, learner cognition, behavioral engagement, and performance outcomes. Specifically, the study incorporates three key technology perception variables—perceived usefulness, perceived ease of use, and trust—alongside writing self-efficacy and AI usage intensity, and models their interrelationships within a second-order structural equation framework. Empirical data were collected from 572 Chinese EFL postgraduate students who regularly employ generative AI tools in English academic writing tasks. This design enables a systematic examination of how learners' perceptions of AI technologies shape their writing self-efficacy and patterns of AI use, and how these factors, in turn, contribute to the development of AI-associated writing anxiety and influence writing performance.

Guided by this framework, the study addresses two central research questions: (1) How do perceived usefulness, perceived ease of use, and trust relate to writing self-efficacy in AI-assisted academic writing contexts? (2) Through what structural pathways do technology perception variables and writing self-efficacy influence AI-associated academic writing anxiety and subsequent performance outcomes? By engaging these questions, the study aims to advance theoretical understandings of technology-enhanced language learning in two important ways. First, it offers a refined conceptualization of writing anxiety as a systemic and interaction-driven phenomenon situated within AI-mediated learning environments. Second, it elucidates the interconnected roles of cognitive, affective, and technological factors in shaping learners' experiences and outcomes.

From a practical perspective, the findings are expected to inform both pedagogical practices and AI tool design. For educators, the results provide insights into how instructional interventions can enhance students' self-efficacy and optimize AI use while reducing anxiety. For developers, the study highlights the importance of usability, perceived value, and trust in shaping user experience and emotional responses. It should be noted, however, that the study does not claim to establish definitive causal relationships; rather, it offers a theoretically grounded account of the structural associations through which writing anxiety can be understood within a broader human–AI interaction system.

### 1.2 Literature review

Up-to-today, generative AI systems have shifted writing technologies from assistive feedback tools to real-time content generators (Li and Hafner, 2022; Shi and Aubrey, 2025). Unlike earlier automated writing evaluation (AWE) systems that primarily provided post-hoc correction (Warschauer and Ware, 2006), large language model–based tools now participate in ideation, structural planning, drafting, and linguistic refinement (Liu et al., 2026; Stevenson, 2016). This shift fundamentally alters the distribution of cognitive labor in writing (Sheeba Sisly et al., 2026). Rather than merely reducing surface-level error correction, generative AI externalizes portions of semantic formulation and rhetorical organization (Kasneci et al., 2023). Educational technology research has documented improvements in drafting efficiency, linguistic accuracy, and revision quality in AI-assisted contexts. However, emerging scholarship also notes unintended consequences, including over-reliance, cognitive offloading, reduced deep processing, and uncertainty regarding authorship boundaries (Zhai et al., 2024). These findings suggest that AI-supported writing environments constitute complex socio-technical systems in which cognitive gains may coexist with affective instability. What needs special attention is that, despite increasing empirical attention to AI adoption in higher education, most studies foreground behavioral intention, usage patterns, or performance enhancement, leaving affective consequences comparatively underexplored (Choudhury and Shamszare, 2023). In high-stakes academic writing contexts, where originality and intellectual ownership are central, AI co-production introduces evaluative tensions that may generate novel forms of anxiety distinct from traditional language-based apprehension (Carr, 2010; Biondi-Zoccai et al., 2025; Wang et al., 2024; Choudhury and Shamszare, 2023; Bai and Yang, 2025).

Data shows that writing anxiety has long been conceptualized as a multidimensional construct encompassing cognitive worry, somatic tension, and avoidance behaviors (Daly and Miller, 1975; Horwitz, et al., 1986; Cheng, 2004; Nugraheni, 2023; Cheng et al., 2026). Empirical studies consistently demonstrate negative associations between writing anxiety and performance, often mediated by self-efficacy (Daly and Miller, 1975; Horwitz et al., 1986; Heidarzadi et al., 2022). Intervention research has focused on pedagogical feedback (McGee, 2019; Tsao et al., 2017; Guo, 2018), strategy instruction, and confidence-building mechanisms (Heidarzadi et al., 2022). However, traditional writing anxiety frameworks were developed in pre-AI writing ecologies (Woodrow, 2011; Wang et al., 2021). Anxiety sources were primarily attributed to linguistic competence, evaluative pressure, task difficulty, or perfectionism (Rahim and Gregory, 2025). These models assume that the learner remains the primary producer of textual content (Busse et al., 2023). In AI-assisted environments, this assumption no longer fully holds. Generative AI introduces additional appraisal dimensions: system opacity, reliability uncertainty, ethical ambiguity, perceived skill displacement, and originality threat. These factors are technological rather than purely linguistic in nature (Ugur and Dursun, 2025). Existing writing anxiety scales do not explicitly account for such technology-mediated evaluations, suggesting a potential conceptual gap when applied to AI-supported academic writing contexts (Li et al., 2013; Guo and Qin, 2010; Asio, 2020; Cheng, 2004; Daly and Miller, 1975). For instance, the Technology Acceptance Model (TAM) posits that perceived usefulness and perceived ease of use predict behavioral intention and system adoption (Zamri et al., 2026). TAM has been widely applied in educational technology research, including studies of AI-based tools (Davis, 1989). Extensions of TAM have incorporated variables such as trust and perceived risk to better explain adoption behavior. However, TAM primarily explains use, not emotional consequence (Akbarini, 2024). Anxiety is typically treated as an external antecedent or peripheral moderator rather than an endogenous outcome embedded within the technology–cognition system. As generative AI becomes integrated into high-stakes academic tasks, emotional responses may not merely precede adoption but may dynamically emerge from ongoing interaction with the technology (Pawlak et al., 2025). This limitation suggests the need to reconceptualize anxiety not as a background variable but as a structurally positioned outcome within AI-supported learning systems.

Recent educational technology scholarship emphasizes the importance of modeling affective processes alongside cognitive and behavioral variables (Schneider et al., 2021; Makransky and Petersen, 2021; Tan et al., 2025). In AI-assisted academic writing, learners simultaneously evaluate: (1) the utility of the tool; the effort required to operate it; the credibility and trustworthiness of its outputs; and the implications for personal competence and intellectual ownership (Davis, 1989). These evaluative processes resemble cognitive appraisal mechanisms that may activate emotional responses, including anxiety (Li and Hafner, 2022). Importantly, such anxiety may not only stem from linguistic difficulty but from perceived integrity threat or skill displacement. Yet empirical modeling of these interrelations remains limited. Few studies integrate technology cognition variables, self-efficacy, usage intensity, and performance outcomes into a unified structural model capable of explaining how AI perception translates into emotional experience and subsequently influences writing performance (Putthidech et al., 2025). Addressing this gap requires an integrative framework that situates anxiety within a dynamic human–AI interaction system rather than treating it as a static trait or isolated psychological state.

Despite the rapid incorporation of generative AI into academic writing practices, there remains a lack of theoretically grounded models that explain how technology-related cognitive appraisals translate into affective outcomes within AI-supported writing environments (e.g., Gámez-Guadix and Mateos-Pérez, 2026). Much of the existing literature on writing anxiety predates the emergence of generative AI and tends to conceptualize anxiety primarily as a linguistically driven phenomenon. In contrast, technology acceptance frameworks focus predominantly on adoption and usage behaviors, often overlooking the emotional consequences that arise in contexts of human–AI co-creation (Liu et al., 2026). As a result, the mechanisms through which perceptions such as usefulness, ease of use, trust, and integrity-related threat interact with writing self-efficacy and AI usage intensity to shape both anxiety and performance outcomes remain insufficiently understood (Downey et al., 2025). This absence of an integrated structural perspective limits the field's ability to explain the seemingly paradoxical role of AI, which may alleviate writing anxiety in some cases while exacerbating concerns related to originality and competence in others. Consequently, both theoretical development and the evidence-based design of AI-supported writing systems are constrained.

Against this backdrop, the present study adopts an integrative analytical framework while also recognizing its empirical and methodological boundaries. The use of cross-sectional survey data from Chinese EFL postgraduate students precludes strong causal inference and may limit the generalizability of the findings across different educational and cultural contexts. Furthermore, reliance on self-reported measures—particularly with regard to writing performance—introduces the possibility of subjective bias.

Notwithstanding these limitations, the study makes several contributions to the emerging field of AI-assisted language learning. First, it reorients the research focus from technological efficacy to learners' affective experiences, foregrounding writing anxiety as a central construct. Second, it advances an integrated framework that links technology-related perceptions, learner cognition (in particular, perceived control via self-efficacy), and emotional outcomes within a unified structural model. Third, it incorporates integrity-related threat as a salient dimension in understanding learners' emotional responses to AI use, thereby extending existing models that have largely neglected this factor. These contributions hold practical relevance for educators, curriculum designers, and researchers engaged in AI-supported writing instruction. By offering a systematic account of how learners' perceptions of AI tools are associated with both emotional responses and performance-related outcomes, the study provides a conceptual foundation for developing more psychologically informed and pedagogically effective approaches to integrating generative AI into academic writing contexts.

## 2 Theoretical framework

The rapid integration of generative artificial intelligence into academic writing has transformed writing from an individual cognitive activity into a human–AI co-regulated process (Li and Hafner, 2022; Mtotywa et al., 2026). In such environments, learners do not merely use technological tools; rather, they continuously evaluate their functionality, reliability, and implications for authorship and competence. Emotional responses, therefore, should be conceptualized as outcomes embedded within this human–technology interaction system rather than as stable personal traits (Goerke et al., 2024). These evaluations are not only cognitive but also affective, as they may shape learners' emotional experiences during writing.

This study is built upon a central theoretical proposition: in AI-assisted academic writing, learners' emotional responses do not arise directly from technology itself, but from how technological affordances are cognitively appraised and translated into perceived control over writing tasks. In other words, technology influences anxiety as a plausible indirect pathway, based on observed associations as it reshapes learners' sense of competence, uncertainty, and authorship legitimacy. Guided by this proposition, the proposed model integrates technology cognition → perceived control (self-efficacy) → emotional outcome (anxiety) → performance into a coherent explanatory chain. This framework conceptualizes anxiety not as an isolated outcome, but as an emergent response resulting from the dynamic interaction between human cognition and AI-mediated writing processes.

The Technology Acceptance Model (TAM) conceptualizes perceived usefulness (PU) and perceived ease of use (PEOU) as key cognitive evaluations that shape users' interaction with technological systems (Davis, 1989). PU reflects the extent to which learners perceive AI tools as beneficial for improving writing outcomes, whereas PEOU represents the degree to which such tools are perceived as easy to operate. In AI-assisted academic writing contexts, these evaluations function as initial appraisals of technological affordances. However, TAM primarily explains technology adoption and usage behavior, rather than emotional consequences. In high-stakes academic writing, additional evaluative dimensions emerge, particularly those related to uncertainty and epistemic reliability. Trust, therefore, is incorporated as a complementary construct, reflecting learners' confidence in the accuracy, credibility, and ethical safety of AI-generated content (McKnight et al., 2002). Unlike PU and PEOU, which focus on functional and operational aspects, trust captures the perceived legitimacy of integrating AI into academic authorship (Mayer et al., 1995). Taken together, PU, PEOU, and trust are conceptualized as technology-related cognitive appraisals. Importantly, these variables are not assumed to directly determine emotional outcomes; rather, they provide the evaluative basis upon which subsequent psychological processes unfold.

Rather than exerting direct effects on writing self-efficacy, PU and PEOU are conceptualized as antecedent cognitive appraisals that shape learners' engagement with AI tools. Drawing on social cognitive theory, self-efficacy is more likely to develop through mastery experiences and perceived control rather than abstract evaluations of technology. Therefore, PU and PEOU are expected to influence writing self-efficacy indirectly, for instance through increased usage, reduced cognitive barriers, and enhanced perceptions of controllability during human–AI interaction. In this sense, technology perception variables do not constitute competence beliefs per se; instead, they function as enabling conditions that may facilitate or constrain the development of such beliefs. Accordingly, the present study reframes the relationship between technology cognition and self-efficacy as associative and potentially mediated, rather than direct and deterministic.

To explain how technology cognition relates to emotional experience, the present study draws on social cognitive theory, which emphasizes the role of self-efficacy as a central mechanism of perceived control (Lazarus, 1991). Writing self-efficacy refers to learners' beliefs in their capability to successfully perform academic writing tasks (Pajares, 2003). Extensive research has demonstrated that self-efficacy is closely associated with both emotional regulation and performance outcomes (Kapil and Hadwin, 2026). In the context of AI-assisted writing, self-efficacy is not assumed to be directly determined by technology perceptions such as PU or PEOU. Instead, it is conceptualized as emerging from learners' interaction with AI tools, including their perceived ability to effectively integrate AI support into their writing process. Technology cognition may be associated with self-efficacy indirectly, for instance by facilitating engagement, reducing operational barriers, or enhancing perceptions of controllability. However, such relationships are treated as associative rather than strictly causal. By positioning writing self-efficacy as an index of perceived control, this study establishes a critical link between technology-related cognition and emotional outcomes. This conceptualization is consistent with the broader view that emotional responses are shaped not only by external conditions but by individuals' perceived capacity to manage those conditions.

Appraisal-based emotion theory posits that anxiety arises when perceived demands exceed perceived coping resources (Lazarus, 1991). In AI-assisted academic writing contexts, perceived demands may include not only linguistic challenges but also technology-related uncertainties, such as the reliability of AI outputs, the risk of over-reliance, and concerns regarding academic integrity (Alenezi et al., 2026). Within this framework, writing anxiety is conceptualized as an outcome of cognitive appraisal processes involving both task-related and technology-related evaluations. Writing self-efficacy, as a representation of perceived control, is expected to be negatively associated with anxiety (Li, 2022). In contrast, integrity-related threat—defined as concerns about authorship legitimacy, originality, and skill displacement—is conceptualized as a distinct source of perceived demand that may be positively associated with anxiety (Frenkenberg and Hochman, 2025). Trust in AI systems is also expected to play a role in emotional regulation, as higher levels of trust may reduce uncertainty-related threat appraisal. However, rather than assuming direct causal effects, the present study treats these relationships as theoretically informed associations that require empirical examination.

In addition to cognitive and affective variables, AI usage intensity is incorporated as a behavioral dimension of human–AI interaction. Unlike traditional TAM frameworks, which treat usage as an outcome of technology acceptance, this study conceptualizes usage intensity as a contextual factor that may be associated with both cognitive and emotional processes. Frequent interaction with AI tools may be associated with increased familiarity and perceived fluency, which could relate to higher perceived control. At the same time, intensive usage may also be associated with heightened awareness of dependency or ethical concerns. Therefore, AI usage intensity is positioned as a potential intermediary variable linking technology cognition, self-efficacy, and anxiety within a broader interaction system.

**Figure 1:**
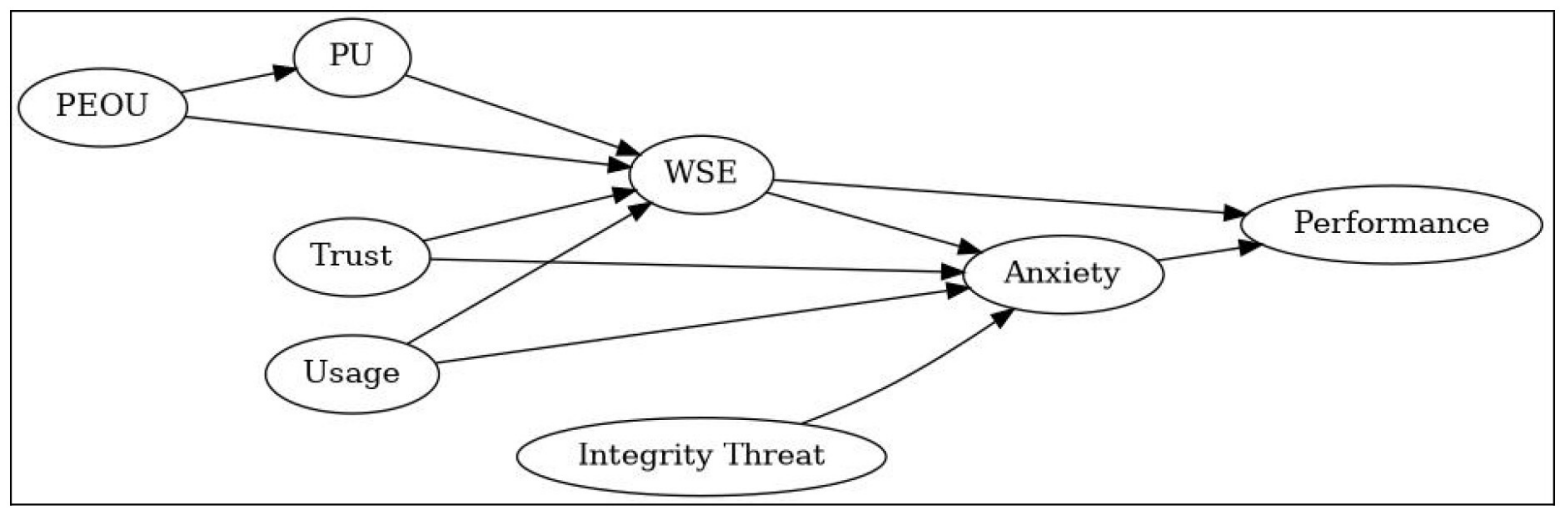
Conceptual model of the TEP framework. PU, perceived usefulness; PEOU, perceived ease of use; WSE, writing self-efficacy; ANX, writing anxiety. Arrows represent theoretically informed associations examined in this study.

Based on the above theoretical considerations, this study proposes a Technology–Emotion–Performance (TEP) framework. In this framework, technology cognition (PU, PEOU, and trust) constitutes the initial appraisal layer; writing self-efficacy represents perceived control; AI usage intensity reflects behavioral engagement; and writing anxiety is conceptualized as an appraisal-based emotional outcome. Writing performance is included as a downstream variable associated with both cognitive and affective processes. Rather than specifying a strictly causal chain, the model represents a theoretically grounded system of associations among these variables. This approach acknowledges the limitations of cross-sectional data while still allowing for the examination of structured relationships informed by prior theory. By situating writing anxiety within a human–AI interaction system, the proposed framework extends existing research in two important ways. First, it conceptualizes anxiety as an endogenous outcome emerging from technology-mediated appraisal processes, rather than as an external or background variable. Second, it integrates cognitive, affective, and behavioral dimensions into a unified model, providing a more comprehensive account of academic writing in AI-assisted contexts. The proposed relationships are illustrated in Figure 1.

The model in Figure 1 delineates a set of interrelated pathways through which technology-related perceptions, learner cognition, and affective responses jointly shape academic writing performance in AI-assisted contexts. And it suggests that the potential benefits of AI-assisted writing tools for performance are contingent upon a balanced interplay of cognitive, behavioral, and affective factors. In particular, improvements in performance are more likely when AI use is positively associated with learners' confidence and is negatively associated with anxiety—conditions that depend critically on both trust in the technology and the absence (or mitigation) of perceived threats to academic integrity.

## 3 Method

This research intends to employ structural equation modeling (SEM) to estimate multiple causal paths, integrating the use of generative AI writing tools, technological anxiety, integrity threats, innovation self-assessment, and anxiety into a unified theoretical network. This approach distinguishes direct effects from potential mediations, revealing the mechanistic differences within the “AI-anxiety” black box. In light of this, this study samples 572 Chinese EFL Master's Students who need to publish English academic papers. Based on the theoretical framework integrating SCT and CAT, we construct and validate a chain model: “use of generative AI writing tools → technological anxiety/integrity threats/innovation self-assessment → psychological anxiety.” The aim is to provide precise evidence and strategic leverage for teaching interventions in college English academic writing, AI ethics education, and the human-centered design of AI tools.

### 3.1 Participants

Participants were 572 Chinese EFL Master's students enrolled in research-oriented postgraduate programs at comprehensive universities. All participants had prior experience using generative AI tools for English academic writing tasks, including drafting, revision, and language refinement. Those Participants represented multiple disciplinary backgrounds, including humanities, social sciences, and STEM fields. Inclusion criteria required active engagement in English-medium academic writing and self-reported use of AI writing tools within the past six months. Participation was voluntary and anonymous. Ethical approval was obtained from the relevant institutional review board. All participants were provided with a detailed explanation of the study's purpose, procedures, potential risks, and benefits. The confidentiality of the participants has been maintained throughout the research process. Informed consent was obtained from all the participants involved in the study, including their consent for paper publication based on the collected data. All the data collected have been anonymized, and any identifying information has been securely stored and will be destroyed after a specified period. The research design and methodology were carefully considered to minimize any potential harm or discomfort to the participants. Any unforeseen issues or concerns that arose during the study were addressed promptly and in accordance with ethical best practices.

### 3.2 Measures

This study employs the confirmatory quantitative research paradigm, utilizing structural equation modeling to examine the theoretical framework of “EFL researchers' academic writing anxiety during generative AI-assisted writing.” The model integrates the “Technology Acceptance Model (TAM)” proposed by Davis (1989) and the “Cognitive-Emotional-Behavioral Model” by Lazarus (1991), incorporating the external technological context of “generative AI writing tool usage” to investigate the formation mechanisms of academic writing anxiety in EFL researchers during the process of writing English academic papers.

The model establishes a second-order latent variable system comprising 7 latent variables and 23 manifest variables (items). The first-order latent variables primarily include: Perceived Usefulness (PU), referring to researchers' subjective beliefs about AI tools' impact on writing efficiency, language quality, and standardization; Perceived Ease of Use (PEOU), indicating subjective evaluations of AI tool operation and learning difficulty; Affective Writing Anxiety (AWA), describing researchers' emotional responses such as tension, fear, and avoidance tendencies during writing tasks; Writing Self-Efficacy (WSE), measuring researchers' confidence in their English academic writing abilities; AI Tool Usage Anxiety (AUA), encompassing researchers' perceptions of threats like “skill displacement,” “devaluation of originality,” and “academic ethical uncertainty” when using generative AI tools; AI Tool Trust (AT), reflecting researchers' confidence in the academic accuracy and ethical safety of AI-generated content; and Writing Performance (PERF), comprising self-rated first draft quality, satisfaction with supervisor feedback, and submission willingness.

All constructs were measured using established scales adapted to the context of AI-assisted academic writing. Items were rated on a Likert-type scale. Technology cognition variables included perceived usefulness (PU), perceived ease of use (PEOU), and trust, reflecting learners' evaluations of AI tools. Writing self-efficacy (WSE) was measured as an index of perceived control over academic writing tasks. Writing anxiety (ANX) captured learners' emotional responses during AI-assisted writing. In addition, integrity-related threat was included to assess concerns related to authorship and ethical use of AI. AI usage intensity was treated as a contextual behavioral variable, reflecting the extent of learners' interaction with AI tools. It was included to examine its association with both perceived control and emotional outcomes. Writing performance was measured through self-reported evaluations of academic writing ability.

### 3.3 SEM specification

Structural equation modeling (SEM) was conducted using maximum likelihood estimation. The analysis proceeded in two stages: Measurement model evaluation (CFA); Convergent validity assessed via standardized loadings (>0.60), composite reliability (CR>0.70), and average variance extracted (AVE>0.50). Discriminant validity assessed through inter-construct comparisons. The direct and indirect effects of Structural model testing were estimated. In addition, mediation effects were also tested using bootstrapped confidence intervals (5,000 resamples), and the Model fit was evaluated using χ^2^/ df, CFI, TLI, and RMSEA. AI usage intensity was modeled as a partial mediator. Overall writing anxiety was specified as a second-order latent variable to capture system-level affective response in AI-assisted writing contexts.

### 3.4 Data analysis

The data analysis utilized structural equation modeling (SEM) and was conducted in two stages. First, descriptive statistics and correlation analyses were executed to examine the relationships among variables. As shown in Table 1, with 572 questionnaires including anxiety-related items (Q26-Q50, Q84-Q91), it is suggested that ANX acts as a second-order factor covering three first-order factors. To document the multidimensional structure of the writing anxiety construct, the specific breakdown of first-order factors, related item numbers, and content descriptions is available in Table 1.

**Table 1:** First-order factors.

First-order factors	Item numbers	Content description
Cognitive anxiety (Cog_ANX)	Q28, Q29, Q30, Q34, Q35, Q45	Worries about terminology, logic, grammar, and language proficiency
Somatic anxiety (Soma_ANX)	Q32, Q38, Q39, Q85, Q86, Q90	Deadline pressure, environmental impact, and emotional fluctuations
Behavioral anxiety (Beh_ANX)	Q40, Q44, Q49, Q84, Q88, Q89	Comparison with others, reactions to feedback, and avoidance behaviors

Table 1 shows the descriptive statistics and reliability estimates for all study variables. This summary offers an initial comprehension of the data distribution and measurement consistency, establishing the foundation for subsequent analyses.

Next, the structural model was examined to assess the associations put forward in the theoretical framework. Model fit was evaluated using several indices, such as the chi-square to degrees of freedom ratio (χ^2^/ df), Comparative Fit Index (CFI), Tucker–Lewis Index (TLI), Root Mean Square Error of Approximation (RMSEA), and Standardized Root Mean Square Residual (SRMR), adhering to commonly accepted thresholds, as displayed in Table 2. In order to validate the higher-order measurement model, Table 2 exhibits the second-order factor loadings, standard errors, z-values, and R^2^ values, which present the correlation matrix among the key variables. These findings provide an initial sign of the associations among technology cognition, self-efficacy, anxiety, and performance, thus guiding the structural model analysis.

**Table 2:** Second-order factor loadings.

First-order factors	Second-order factor loading (λ)	Standard error (SE)	*z*-value	*R^2^*
Cognitive anxiety (Cog_ANX)	0.89	0.03	290.67	0.79
Somatic anxiety (Soma_ANX)	0.92	0.02	46.00	0.85
Behavioral anxiety (Beh_ANX)	0.85	0.04	21.25	0.72

*p* < 0.001.

Table 2 presents the second-order factor loadings of writing anxiety, indicating how the three first-order dimensions—cognitive anxiety, somatic anxiety, and behavioral anxiety—contribute to the higher-order construct. As shown in Table 2, all three first-order factors load strongly onto the second-order factor, with standardized loadings ranging from 0.85 to 0.92. Somatic anxiety shows the highest loading (λ = 0.92), followed by cognitive anxiety (λ = 0.89) and behavioral anxiety (λ = 0.85), suggesting that all three dimensions are substantial components of the overall anxiety construct. The standard errors are relatively small, and all z-values are large and statistically significant (p < .001), indicating that the loadings are robust and reliably estimated. In addition, the *R*^2^ values range from 0.72 to 0.85, suggesting that a substantial proportion of variance in each first-order factor is explained by the second-order construct. Overall, these results provide strong support for modeling writing anxiety as a higher-order construct composed of cognitive, somatic, and behavioral dimensions.

**Figure 2:**
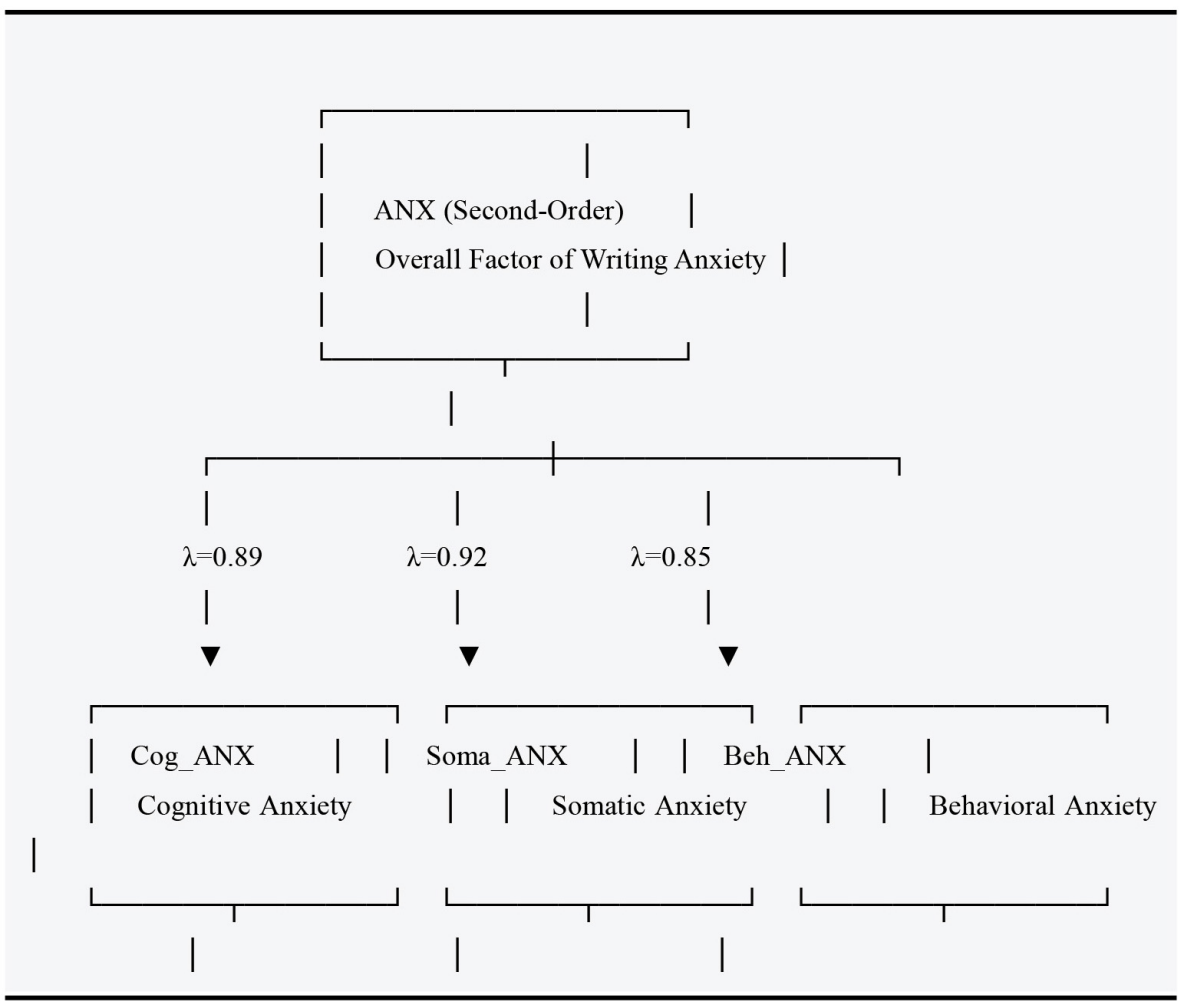
Second-order CFA model diagram.

The data presented in Figure 2 reveals that, the ellipse at the top represents the second-order factor (ANX, overall writing anxiety factor); the three middle ellipses represent the three first-order factors (Cog_ANX, Soma_ANX, Beh_ANX); the rectangles below each first-order factor represent the observed items corresponding to each dimension. λ represents the standardized second-order factor loading, reflecting the correlation strength between each first-order factor and the second-order factor. δ denotes the measurement error of the observed items, and ζ denotes the residual of the first-order factors, which are all included in the model to meet the CFA modeling standards.

The collected data from the survey also reveals that, the Second-Order Model is superior to the First-Order Model, and this superiority is comprehensively reflected in four aspects: theoretical fit, parsimony, reliability, and predictive validity. To justify the theoretical and practical superiority of the second-order model, the specific comparison and advantage explanation are shown in Table 3.

**Table 3:** Comparison between first-order model and second-order model.

Comparison dimension	First-order model	Second-order model	Advantage explanation
Theoretical fit	Three independent factors	Common latent variable ANX explains the correlation	Consistent with the theory of “writing anxiety” as an overall construct
Parsimony	3 factor covariances = 3 parameters	3 second-order loadings = 3 parameters	Same degree of freedom but more explanatory power
Reliability	Reliability calculated for each factor separately	Overall ANX reliability can be calculated	Higher level of consistency
Predictive validity	Separate prediction required for each factor	Prediction with a single ANX score	More concise subsequent analysis

Regarding this superiority, there are several core reasons: Firstly, at the level of construct validity, writing anxiety is a higher-order psychological construct, and the three dimensions (cognitive, somatic, and behavioral anxiety) are its different manifestations, which are not isolated but driven by the overall writing anxiety; Secondly, at the level of variance, the second-order model shows that the three dimensions share 66-79% of the variance, which supports the existence of a common latent variable (ANX); And finally, from the Practical Application level, a single ANX total score can be generated for subsequent regression or SEM (Structural Equation Modeling) analysis, which simplifies the analysis process and improves the efficiency of research.

To further verify the rationality of the second-order model, the first-order CFA model (treating the three anxiety dimensions as independent latent variables) was constructed for comparative analysis. The fit indices of the two models, their differences, and the χ^2^ difference test results are shown in Table 4.

**Table 4:** Comparison of fit indices.

Fit indices	First-order model	Second-order model	Difference
χ^2^ (df)	245.32 (132)	248.67 (134)	Δχ^2^ = 3.35
CFI	0.976	0.974	ΔCFI = −0.002
TLI	0.968	0.971	+ 0.003
RMSEA (90% CI)	0.039 (0.031-0.047)	0.038 (0.030-0.046)	−0.001
SRMR	0.041	0.043	+ 0.002
AIC	12,456.78	12,452.12	−4.66
BIC	12,678.90	12,665.34	−13.56

To further examine whether the second-order structure provided a statistically equivalent fit compared to the first-order model, a chi-square difference test was conducted, and the test suggested that there was an extremely significant chi-square difference between the second-order model and the first-order model. With a slight increase in degrees of freedom, the chi-square value decreases significantly, indicating that the second-order model can fit the data better with a more parsimonious structure. Thus the study concludes that, there is no significant difference in fit between the second-order model and the first-order model (*p* > 0.05). However, the second-order model has obvious advantages: (1) More parsimonious, with lower AIC and BIC values; (2) Stronger theoretical explanatory power, which conforms to the holistic construct of writing anxiety; (3) ΔCFI = −0.002 < 0.01, which meets the equivalence criterion proposed by Cheung and Rensvold (2002), indicating that the fit of the two models is equivalent, and the second-order model is more preferred due to its better theoretical and practical value. It is more parsimonious (lower AIC and BIC) and has stronger theoretical explanatory power, which is more in line with the holistic nature of writing anxiety as a psychological construct.

To conclude, the second-order CFA model of ANX constructed in this study is scientific and reasonable, which not only verifies the multidimensional and holistic characteristics of academic writing anxiety but also provides a reliable measurement tool for subsequent research on the influencing factors, mediation/moderation effects of writing anxiety, and the formulation of targeted anxiety relief strategies for non-English major postgraduates' scientific English writing teaching.

## 4 Results

### 4.1 Descriptive statistics and correlations

Table 2 presents the descriptive statistics and reliability estimates for all study variables, including means, standard deviations, skewness, kurtosis, and Cronbach's α coefficients. Table 3 reports the correlation matrix among the key constructs. These analyses provide an initial overview of data distribution, measurement consistency, and the associations among technology cognition, writing self-efficacy (WSE), writing anxiety (ANX), and writing performance.

As shown in Table 2, all variables demonstrated acceptable levels of skewness (−0.22 to 0.19) and kurtosis (−0.30 to 0.63). These values fall within commonly recommended thresholds, indicating no substantial deviation from normal distribution. Cronbach's α coefficients ranged from 0.80 to 0.84, exceeding the recommended criterion of 0.70 and demonstrating satisfactory internal consistency reliability.

As reported in Table 3, all correlations among the latent constructs were statistically significant and remained below the multicollinearity threshold (*r* < 0.85). This suggests that discriminant validity is acceptable at the preliminary level. The observed correlation patterns were generally consistent with theoretical expectations: technology cognition variables (perceived usefulness, perceived ease of use, and trust) were positively associated with writing self-efficacy and writing performance, and negatively associated with writing anxiety. It should be noted that these results reflect associational relationships rather than causal effects, given the cross-sectional nature of the data.

Common method variance was assessed through a single-factor confirmatory factor analysis. The single-factor model showed acceptable fit (χ^2^/ df = 1.86, CFI = 0.97, RMSEA = 0.038), suggesting that common method bias is unlikely to be a serious threat to the validity of the findings. In addition, collinearity diagnostics indicated that all VIF values were below the recommended threshold of 3.3, further supporting the robustness of the measurement model.

### 4.2 Measurement model: second-order structure of writing anxiety

To capture the multidimensional nature of writing anxiety, a second-order confirmatory factor analysis (CFA) was conducted. As specified in Table 1, writing anxiety (ANX) was conceptualized as a higher-order construct comprising three first-order dimensions: cognitive anxiety, somatic anxiety, and behavioral anxiety. Table 4 reports standardized factor loadings, standard errors, z-values, and explained variance (R^2^). All first-order dimensions loaded strongly on the higher-order construct, with standardized loadings ranging from 0.85 to 0.92, indicating a strong shared variance structure. The corresponding *R*^2^ values (0.72–0.85) further confirm that a substantial proportion of variance in each first-order dimension is accounted for by the higher-order construct. These results support the representation of writing anxiety as a higher-order multidimensional construct, in which cognitive, somatic, and behavioral components reflect related but distinct manifestations of a broader affective state.

Figure 1 illustrates the second-order CFA model, in which the higher-order latent variable (ANX) captures shared variance across the three first-order dimensions, while measurement errors and residual terms are explicitly modeled following standard CFA specifications.

### 4.3 Model comparison and justification of the second-order structure

To further examine the appropriateness of the second-order representation, a second-order CFA model was estimated, and its fit indices are reported in Table 5. As presented in Table 5, the model demonstrated a good fit to the data, with χ^2^/ df = 1.86, CFI = 0.97, TLI = 0.96, RMSEA = 0.038, and SRMR = 0.043. All indices meet or exceed commonly accepted thresholds, indicating a strong correspondence between the hypothesized measurement structure and the observed data. Specifically, the χ^2^/ df value is below the recommended cutoff of 3.0, while both CFI and TLI exceed 0.95, suggesting strong incremental fit. In addition, RMSEA and SRMR values are below 0.05, indicating a close approximation of the model to the data.

**Table 5:** Model fitting indicator data.

Indicator name	Indicator value
χ^2^/ df	1.86
CFI	**0.97**
TLI	0.96
RMSEA	0.038
SRMR	0.043

### 4.4 Structural model and explained variance

Table 6 presents the R^2^ values of endogenous constructs in the structural model, reflecting the explanatory power of the proposed framework, including PEOU, PU, trust, and integrity threat.

**Table 6:** Variance explained (*R*^2^) in the structural model.

Endogenous variable	Predictor(s)	R^2^	Effect size	Interpretation
Writing self-efficacy	Technology-related cognitive appraisals	0.58	Substantial	A considerable proportion of perceived control is associated with technology-related cognitive appraisals
Writing Anxiety	Writing self-efficacy	0.31	weak	A weak association exists between perceived competence and emotional response
Writing performance	Writing self-efficacy + Writing anxiety	0.49	Moderate	Nearly half of the variation in performance is associated with the cognitive–affective mechanism specified in the model

**Figure 3:**
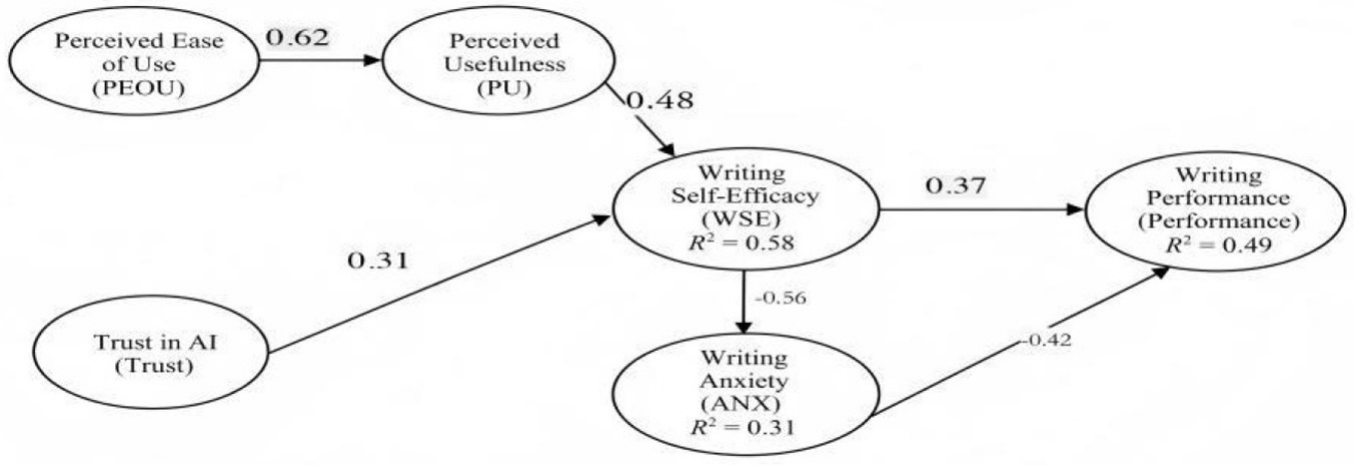
Structural model with standardized path coefficients.

Effect size classification: According to Sarstedt et al. (2017), *R*^2^ values of 0.75, 0.50, and 0.25 can be interpreted as substantial, moderate, and weak explanatory power respectively. *R*^2^ values reflect the full structural model including all exogenous predictors shown in Figure 3.

Writing self-efficacy was explained by technology-related cognitive appraisals (including perceived usefulness, perceived ease of use, and trust), yielding an *R*^2^ value of 0.58. This indicates that a substantial proportion of variance in perceived writing competence is associated with technology-related cognitive evaluations. Writing anxiety was jointly explained by writing self-efficacy and additional technology-related perceptions (e.g., trust and integrity-related threat), with an R^2^ value of 0.31. This reflects a moderate level of explanatory power for affective responses within the proposed model. While writing performance was explained by both writing self-efficacy and writing anxiety, yielding an *R*^2^ value of 0.49, indicating that approximately half of the variance in writing performance is associated with the combined cognitive–affective variables specified in the model. According to commonly applied guidelines in structural equation modeling (e.g., Sarstedt et al., 2017), these values indicate moderate to substantial explanatory power, particularly for writing self-efficacy and writing performance.

A sequential indirect pathway involving both self-efficacy and anxiety was also supported. Technology cognition variables were associated with higher self-efficacy, which was in turn associated with lower anxiety, and subsequently with better performance. The confidence intervals for these indirect pathways excluded zero. It is important to emphasize that these findings reflect plausible indirect associations rather than definitive mediation effects, given the cross-sectional design of the study.

### 4.5 Indirect effects (associational pathways)

Bootstrapping analysis (5,000 resamples, 95% bias-corrected confidence intervals) was conducted to examine indirect associations among variables. The results indicated that technology cognition variables were indirectly associated with writing performance through writing self-efficacy and writing anxiety. Specifically, higher levels of perceived ease of use, perceived usefulness, and trust were associated with higher writing self-efficacy, which was associated with lower writing anxiety, and in turn with higher writing performance. The corresponding confidence intervals did not include zero. These findings suggest the presence of statistically meaningful indirect associations within the proposed structural framework. However, given the cross-sectional design of the study, these pathways should be interpreted as associative rather than causal or temporal effects.

### 4.6 Structural model results

Figure 3 presents the structural model with standardized path coefficients, illustrating the relationships among key constructs.

Figure 3 visually presents the structural connections between the variables, aiding in a more comprehensive understanding of the model. The structural model in Figure 3 matches the conceptual framework from Figure 1, enabling an empirical analysis of the theoretically defined relationships. It compiles the results of the structural model, such as standardized path coefficients and significance levels. These results directly tackle the research questions by exploring the relationships suggested in the theoretical framework.

As illustrated in Figure 3, writing self-efficacy (WSE) is negatively associated with writing anxiety (ANX), suggesting that higher perceived control is related to lower levels of anxiety. This pattern indicates a consistent cognitive–affective association, where perceived competence co-occurs with reduced emotional tension in academic writing contexts. Perceived ease of use (PEOU) is positively associated with perceived usefulness (PU), which in turn shows a positive association with writing self-efficacy. This sequential pattern suggests that favorable cognitive evaluations of AI tools are linked to higher perceived control in writing, although these relationships are interpreted as associative rather than causal.

In addition, trust in AI tools is positively associated with writing self-efficacy and shows a negative association with writing anxiety, indicating that greater trust is related to both enhanced perceived competence and reduced emotional burden. Integrity-related threat, by contrast, is positively associated with writing anxiety, suggesting that perceived ethical or reliability concerns are linked to heightened emotional strain during AI-assisted writing.

Regarding performance outcomes, writing anxiety is negatively associated with writing performance, whereas writing self-efficacy shows a positive association with performance. These patterns indicate that both cognitive control beliefs and emotional responses are meaningfully related to students' writing outcomes. These results suggest a coherent **cognitive–affective associative mechanism**, in which technology-related perceptions are linked to writing outcomes through perceived control (self-efficacy) and emotional responses (anxiety). Rather than implying direct causality, the findings highlight a structured pattern of relationships among cognitive appraisals, affective states, and performance outcomes in AI-assisted academic writing.

The findings provide empirical support for the theoretical proposition that “In AI-assisted academic writing, anxiety is not directly produced by technology itself, but is associated with how technological affordances are cognitively appraised and translated into perceived control.” Within this framework, the reported R^2^ values further highlight the explanatory relevance of perceived control and emotional response variables in understanding writing performance. At the same time, these findings should be interpreted with caution. Given the cross-sectional nature of the data, causal inferences cannot be established, and alternative explanations or reciprocal relationships remain possible. Future research employing longitudinal or experimental designs is needed to further validate the proposed mechanisms. However, it should be noted that direct paths from technology perception variables to writing anxiety were not the primary focus of the present model. Therefore, the results should not be interpreted as evidence that such direct associations are absent.

## 5 Discussion and conclusion

The present study set out to examine how technology-related perceptions, learner-level regulation, and behavioral engagement are associated with academic writing anxiety in AI-assisted contexts. By integrating constructs from technology acceptance, social cognitive theory, and appraisal-based emotion theory, the proposed framework provides a structured account of how cognitive evaluations of AI tools may relate to emotional experience and writing outcomes. The results offer several theoretically meaningful insights, while also requiring cautious interpretation given the cross-sectional nature of the data. However it should be noted that the directional paths specified in the model are theory-driven and do not imply causal ordering.

A central implication of the findings is that academic writing anxiety in AI-assisted environments can be more appropriately understood as a technology-mediated appraisal outcome rather than solely a function of linguistic competence or task difficulty. The observed associations between technology cognition variables (e.g., perceived usefulness, ease of use, and trust), self-efficacy, and anxiety suggest that learners' emotional experiences are embedded within a broader system of human–AI interaction. Rather than treating anxiety as an isolated psychological trait, the results support a perspective in which emotional responses are linked to how learners interpret both the demands of the writing task and the affordances of AI tools. In this sense, anxiety appears to reflect a combined appraisal of task-related challenges and technology-related uncertainties, including issues of reliability, control, and authorship legitimacy. This interpretation extends prior writing anxiety research by incorporating technology-mediated appraisal processes into the conceptualization of affect in academic writing.

The findings also highlight writing self-efficacy as a key variable associated with lower levels of anxiety and higher levels of writing performance. Consistent with appraisal-based accounts of emotion, this pattern suggests that perceived control may play an important role in shaping emotional experience in AI-assisted writing contexts. Importantly, the present study does not support a simplistic interpretation in which technology perceptions directly translate into competence beliefs. Instead, the results indicate that perceived usefulness and ease of use are associated with self-efficacy, which in turn is strongly related to anxiety. This pattern is consistent with the view that technology-related evaluations may contribute to perceived control indirectly, for example by facilitating engagement or reducing operational barriers. However, given that direct effects between technology perception variables and anxiety were not explicitly modeled, these relationships should be interpreted as indicative of a plausible indirect pathway rather than definitive evidence of full mediation. This interpretation also helps explain why AI assistance may be associated with both reduced and heightened anxiety in different contexts. If interaction with AI tools is associated with increased perceived control, anxiety may be attenuated; conversely, if such interaction undermines confidence or introduces uncertainty, anxiety may persist or increase.

Another important finding concerns the role of trust and integrity-related threat. Trust was negatively associated with anxiety, suggesting that confidence in the reliability and legitimacy of AI-generated content may be linked to reduced uncertainty-related concern. At the same time, integrity-related threat emerged as a positive predictor of anxiety, indicating that concerns about authorship, originality, and ethical boundaries constitute a distinct dimension of emotional experience in AI-assisted writing. Taken together, these results suggest that emotional responses in AI-supported environments are shaped not only by perceptions of usefulness or ease, but also by how learners evaluate the epistemic and ethical implications of using AI tools. This expands traditional models of writing anxiety by introducing identity- and integrity-related appraisal processes as relevant factors. It is important to note, however, that these associations do not establish causal direction. While the theoretical framework assumes that such appraisals may precede emotional responses, alternative interpretations (e.g., that anxious learners are more sensitive to integrity concerns) cannot be ruled out. Longitudinal or experimental research would be required to disentangle these possibilities.

The inclusion of AI usage intensity provides additional insight into the behavioral context of AI-assisted writing. The observed associations suggest that usage is linked to both cognitive and affective variables, supporting the view that emotional experience is shaped through ongoing interaction rather than static evaluation. At the same time, the findings do not indicate a uniform effect of usage. Increased interaction with AI tools may be associated with greater familiarity and perceived fluency, but may also coincide with heightened awareness of dependency or ethical concerns. This suggests that usage intensity may function as a contextual factor within a dynamic system, rather than as a simple predictor of either positive or negative outcomes.

As for the implications for theory, the present study contributes to the literature in several ways. First, it extends technology acceptance research by incorporating affective outcomes as endogenous components of the model, rather than treating them as external variables. Second, it refines writing anxiety theory by demonstrating that, in AI-assisted contexts, anxiety may be linked to technology-mediated appraisal processes in addition to linguistic factors. Third, it highlights the importance of perceived control (self-efficacy) as a connecting mechanism between cognition and emotion, while avoiding assumptions of direct causal effects. Importantly, these contributions should be understood as theoretically informed interpretations of observed associations, rather than definitive claims about underlying causal mechanisms. The proposed framework is intended as a basis for further empirical testing rather than a conclusive model.

Several limitations should be acknowledged. This study is based on a cross-sectional design, which limits the ability to draw causal inferences. Although the structural model specifies directional relationships among variables, these paths should be interpreted as theoretically informed associations rather than evidence of causality. Establishing causal mechanisms would require longitudinal or experimental designs that can track changes over time or manipulate key variables. Future studies employing longitudinal or experimental designs are needed to examine how these relationships evolve and whether the proposed pathways hold under causal testing. In addition, the reliance on self-reported data may introduce common method bias, despite the statistical checks conducted. The sample, consisting of Chinese EFL postgraduate students, may also limit generalizability to other populations or educational contexts. Finally, writing performance was self-rated rather than objectively assessed, which may affect the interpretation of performance-related findings. Future research may address these limitations by incorporating multi-source data, objective writing measures, and cross-cultural samples. It should be noted that the findings should be interpreted cautiously; Future research should simultaneously test both direct and indirect effects. Most importantly, it would also be valuable to explicitly test competing models (e.g., including direct paths from technology perception to anxiety) to further clarify the structure of relationships proposed in this study. Future research is therefore needed not only to replicate these associations across contexts, but also to examine their temporal and causal dynamics using more robust research designs.

When considered together, the findings are best interpreted as indicating a structured pattern of associations that is consistent with an appraisal-based account of writing anxiety, rather than as evidence of a unidirectional or fully mediated causal mechanism. The findings also suggest a structured pattern of relationships in which technology cognition is linked to perceived control, which in turn is associated with emotional experience and self-reported writing performance. Importantly, the study does not establish causal relationships but instead provides a theoretically informed account of how these variables may be interconnected within a human–AI interaction system. In this sense, writing anxiety is better understood not as an isolated psychological trait, but as an appraisal-based outcome shaped by both task-related demands and technology-related evaluations. Overall, this study provides a foundation for understanding academic writing anxiety in AI-assisted environments and offers a basis for further research into the complex interplay between technology, cognition, and emotion in language learning.

## Data Availability

The original contributions presented in the study are included in the article/supplementary material, further inquiries can be directed to the corresponding author.
